# A new genus and species of Placusini from a high mountain in Mexico

**DOI:** 10.3897/zookeys.640.10911

**Published:** 2016-12-13

**Authors:** Quiyari J. Santiago-Jiménez, Rosny Santiago-Navarro

**Affiliations:** 1 Museo de Zoología, Facultad de Biología-Xalapa, Universidad Veracruzana, Zona Universitaria, Circuito Gonzalo Aguirre Beltrán s/n, Xalapa, Veracruz, C.P. 91090, MÉXICO

**Keywords:** Aleocharinae, Nearctic, Pinus forest, semiochemicals

## Abstract

A new genus and species are described from the Cofre de Perote volcano, in the state of Veracruz, Mexico. Although the new genus is very similar to *Placusa*, it presents tergite VIII completely modified to form a horn, in both females and males, in addition to other differences in mouthparts. A map and illustrations are provided, as well as an identification key to the genera of Placusini. No morphological characters are apparent to separate *Kirtusa* Pace from *Euvira* Sharp in our genus key. The specimens of the new genus were collected using Lindgren and cross traps baited with a mix of semiochemicals: ipsenol, ipsdienol and lanierone.

## Introduction

The tribe Placusini currently includes four genera: *Euvira* Sharp is known from North America to Argentina, including the Antilles (Ashe and Kistner 1989), *Kirtusa* Pace is only known from Ecuador (Pace 2008), *Speiraphallusa* Pace was described from Malaysia (Pace 2013), and *Placusa* Erichson is distributed worldwide (Newton et al. 2000). The genus *Placusa* Erichson, 1837 has been recorded from every zoogeographical region: five species from the Australian region, 24 species are Afrotropical, nine species are Nearctic, 44 Neotropical, 51 Oriental, and 13 Palearctic (Newton, pers. comm.). Even within this genus, some species have a wide range of distribution. *Placusa complanata* Erichson, 1839 has a Holarctic distribution (Erichson 1839, Hamilton 1894), *Placusa tenuicornis* Fauvel, 1878 is found in the Oriental and Australian regions (Fauvel 1878; Bernhauer 1920), and *Placusa pygmaea* Kraatz, 1859 is distributed throughout the Oriental, Afrotropical and Australian regions, though its type locality is in Sri Lanka (= Ceylon) (Kraatz 1859, Fauvel 1903, Cameron 1939, Pace 1992, Pace 2006). The distribution of the latter species is atypical and requires corroboration.

Here, a new genus of Placusini is proposed based on specimens collected on the Cofre de Perote volcano, Veracruz, Mexico. The new genus is very similar to *Placusa*, according to the *Placusa* diagnosis proposed by Klimaszewski et al. (2001), but can be distinguished from *Placusa* and other Placusini based on several morphological differences.

## Materials and methods

From March to May in 2015, 28 specimens of Placusini were collected in the Cofre de Perote volcano, specifically in the locality Agua de los Pescados, Veracruz state, Mexico. The specimens were collected using handmade interception traps, baited with a mix of semiochemicals: ipsenol, ipsdienol and lanierone. Specimens were preserved in ethanol 70%, and later observed and identified using a Stemi DV4 stereoscopic microscope. For the illustrations, photographs were taken using an image processing system (VELAB microscope model VE-633 with Digital LCD model DMS-153). Permanent microscope slides were prepared using the techniques described by Santiago-Jiménez (2010). Habitus photographs were taken through a Nikon SMZ25 stereoscopic microscope. The terminology used here mostly follows Ashe and Kistner (1989), Klimaszewski et al. (2001), and Santiago-Jiménez (2010). The holotype and paratypes were deposited in MUZ-UV—Museo de Zoología, Facultad de Biología Región Xalapa, Universidad Veracruzana, Xalapa, México (Dr. Q. Santiago-Jiménez).

## Taxonomy

### Placukorna

Taxon classificationAnimaliaColeopteraStaphylinidae

Santiago-Jiménez gen. n.

http://zoobank.org/B4A0BE1A-74D5-4454-B325-BC05958876BB

#### Diagnosis.

**Figure 1.**
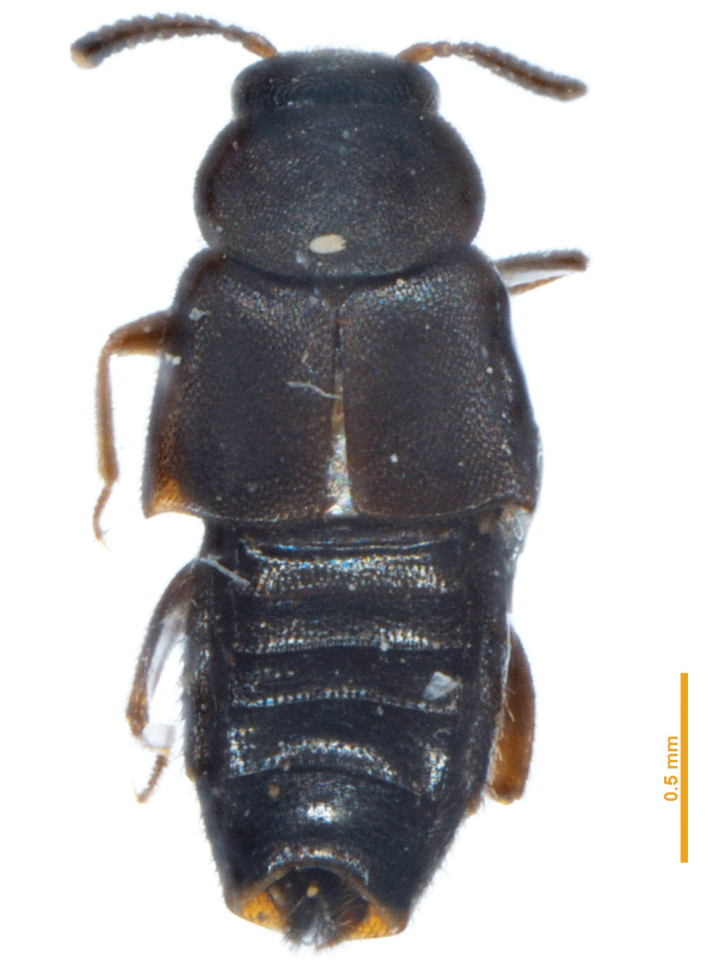
Habitus of *Placukorna ipsa* Santiago-Jiménez, gen. n. and sp. n. (Holotype).

**Figure 2.**
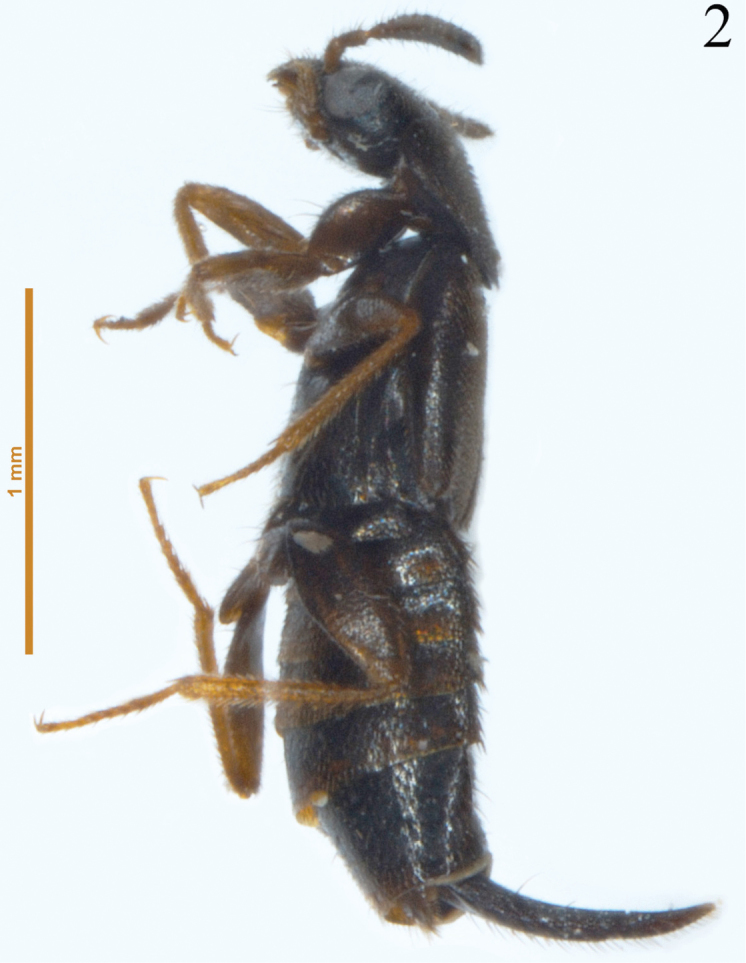
Lateral view of *Placukorna ipsa* Santiago-Jiménez, gen. n. and sp. n. (Holotype).

Body shape fusiform, broad and strongly flattened; head transverse, with a suture between antennal insertions; sensillae on apical margin of epipharynx arranged in a pattern of anterior or a-sensilla, and lateral or ε-sensilla; prementun with medial pseudopore field present, with at least a few pseudopores in an irregular rectangular array, but pseudopores are extended to the lateral pseudopore field; mandibles with a large velvety patch completely occupying the base, composed of nine to eleven transverse rows of large teeth that are reduced in size to the base; labium with short rounded ligula, entire, and not divided; pronotum transverse, approximately 1.5 times wider than long, wider on medial third; mesocoxal cavities not separated by meso- and metaventrite processes; mesoventrite process short with apex acuminate; metaventral process medium-sized, marginate and with apex subobtuse; isthmus not present; tergite VII with lateral margin modified to form a structure-like wall on each side, which apparently provides support between tergite and sternite (only visible because of transparency on slides), and with an apical sclerotized plate attached internally (only visible because of transparency on slides) to receive tergite VIII; tergite VIII modified to form a horn (Figs 1–2), in females and males; spermatheca with small and approximately spherical capsule, median and posterior portion of spermathecal stem (duct) sinuate; process of crista apicalis long, almost straight and parallel to median lobe, rounded at the apex.

#### Description.

Body length 2.5–3.0 mm. Body shape fusiform, broad and strongly flattened; pronotum transverse. Tergite VIII modified to form a horn (Figs 1–2) in both, males and females.

**Figures 3–14.**
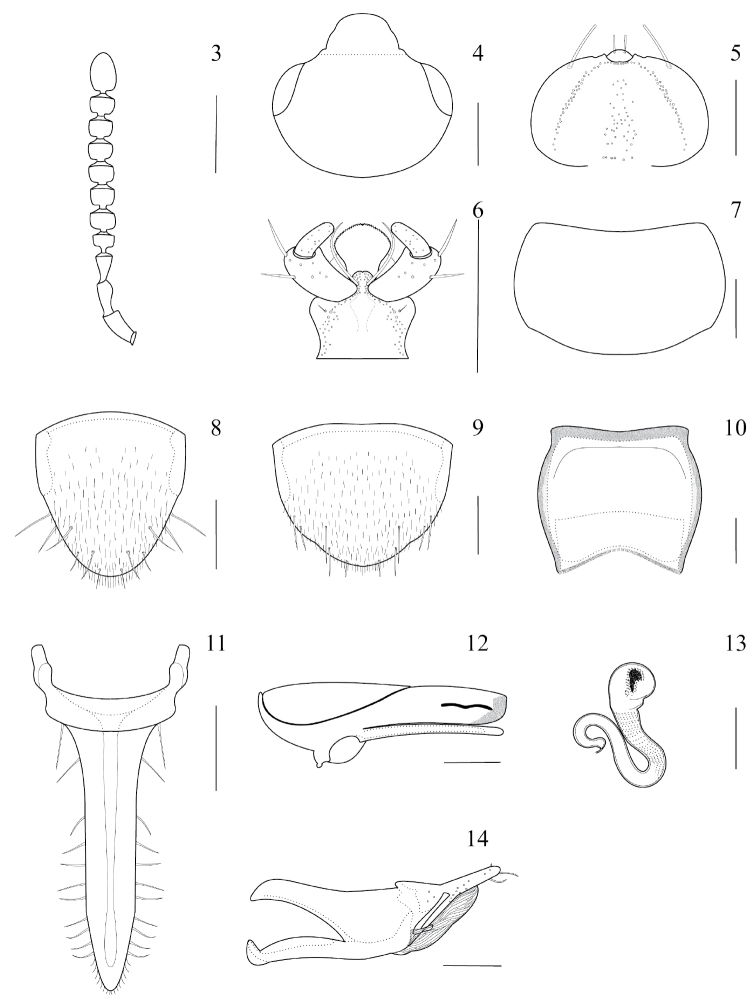
*Placukorna ipsa* Santiago-Jiménez, gen. n. and sp. n. male (**8, 12, 14**) and female (**9, 13**). **3** antenna **4** head **5** epipharynx **6** ligula, prementum, and labial palpi **7** pronotum **8** sternite VIII **9** sternite VIII **10** sternite VII tergite VIII **12** median lobe, lateral view **13** spermatheca **14** paramere, lateral view. **Scale bars: 3–4, 7–11** = 0.2 mm; **5–6, 12–14**, = 0.1 mm.

*Head*. Transverse, with a suture between antennal insertions; surface with coarse punctures densely distributed. Antennomeres 4–10 transverse (Fig. 3). Eyes prominent, occupying almost two thirds of head length. Neck absent (Fig. 4). Infraorbital carina present. Coeloconic sensilla absent (Fig. 3).

*Mouthparts. Labrum*: with 7 setae on each side of the midline; most of the setae on anterior half; with 17–19 sensory pores on each side of midline; sensillae on apical margin of epipharynx, arranged in a pattern of anterior or a-sensilla, and lateral or ε-sensilla (Fig. 5), one on each side of the midline (see Ashe 1984, Santiago-Jiménez 2010); apico-medial margin of epipharynx not modified to setose or with spinose process; basal region of epipharynx with six pores, more or less in one transverse row; medial region of epipharynx with 35–40 pores in an irregular array (Fig. 5); medial region of epipharynx without a multiporose sensory structure on each side of midline; basal region epipharynx without pores on each side to form a transverse row. *Mandibles*: asymmetrical; right mandible with a medial tooth, poorly defined; with serration on apical half of mandibles; with a large velvety patch completely occupying the base, composed of nine to eleven transverse rows of large teeth that are reduced in size to the base; prosthecal setae are not bifurcated on medial area. *Maxilla*: with a row of nine spines (in one specimen only seven were counted) and scarce setae contiguous on apical half of lacinia, basal half almost glabrous, with only five setae; with scarce setae on apical third of galea and two spines, medial and basal third almost glabrous; with pseudopores on the cardo. *Labium*: with short rounded ligula, entire, and not divided. Prementum with two medial setae insertions widely separated; medial pseudopore field present, with at least a few pseudopores in an irregular rectangular array, but pseudopores are extended to the lateral pseudopore field (Fig. 6); lateral pseudopore field composed of one setose pore, and two asetose pores (Fig. 6); with setae on adoral margin of hypoglossa, but without setae on aboral margin. Mentum without reticulate microsculpture on surface; with scarcely distributed pores on mentum, around fourteen on each side of midline; with a pair of macrosetae on each apico-lateral margin, one macroseta is longer than the other is; surface with eight setae on each side of midline. Labial palpi with only two segments.

*Thorax*. Pronotum transverse (Fig. 7), approximately 1.5 times wider than long, wider on medial third; surface finely punctured, moderately dense to dense; without reticulate microsculpture; setae dense on surface; apparently without macrosetae on surface. Scutellum with surface smooth, with some punctures, and moderately covered with short setae. Elytra together slightly wider on apical area, but on basal area slightly wider than pronotum; surface punctured moderately dense; without reticulate microsculpture; setae densely distributed, covering the surface; without macrosetae. Hind wings well developed. Mesocoxal acetabula margined posteriorly. Mesocoxal cavities not separated by meso- and metaventrite processes; mesoventrite process short (approx. 0.26 mm) with apex acuminate; metaventral process medium-sized (approx. 0.35 mm), marginate and with apex subobtuse; isthmus not present. Legs with tarsal formula 4–4–5, each apical tarsus with an empodium, one seta on empodium and a pair of tarsal claws, each claw with a subbasal tooth.

*Abdomen*. Abdomen fusiform (Fig. 1), narrower than elytra, although tergite VIII is modified to form a long horn; tergites with scarce setae; sternites with dense microsetae (Figs 8–9), but slightly less dense than elytra, almost without macrosetae on abdominal segments III-VI except on lateral margins of sternites; tergite VIII modified to form a long horn almost glabrous on dorsal surface, but with long setae on ventral surface, sparsely to slightly densely distributed. Tergite VII (Fig. 10) with lateral margin modified to form a structure-like wall on each side, which apparently provides support between tergite and sternite (only visible because of transparency on slides), and with an apical sclerotized plate attached internally (only visible because of transparency on slides) to receive tergite VIII (Fig. 11). Tergite VII has a small “U” incision on midline to receive the modified tergite VIII.

#### Remarks.

The new genus is very close to *Placusa*; however, it can be distinguished easily by tergite VIII, which is modified completely to form a horn, and the sclerotized lateral internal wall of tergite VII, that apparently supports tergite VIII. Tergite VII has a sclerotized internal plate in the posterior margin that may also support tergite VIII. Moreover, there are some differences in the median lobe of *Placusa* (crista apicalis shorter) and *Placukorna* (crista apicalis longer; Fig. 12) upon comparison. In addition, *Placukorna* shows lateral or ε-sensilla on the epipharynx, the medial field of epipharynx is not flanked by rows of large scales, and prementum with lateral field of pores with pseudopores extended from medial field. The same characters are useful for distinguishing the new genus from other Placusini. Another useful character for distinguishing *Placukorna* from *Placusa* is the absence of spine-like dents on the apex of tergite VIII that are present *Placusa* males.

#### Type species.

*Placukorna ipsa* Santiago-Jiménez sp. n.

#### Etymology.

The genus name is a combination of “*Placusa*” and “*korna*”, from the Greek “Πλαξ” (meaning surface plane) and “κόρνα” (meaning horn), respectively.

#### Gender.

Neuter.

#### Habitat.

Specimens were found in Lindgren and cross traps baited with ipsenol, ipsdienol and lanierone in a mixed pine forest. The forest is composed of *Pinus pseudostrobus*, *Pinus montezumae* and *Pinus patula*, located around 3090 m a.s.l.

**Figure 15.**
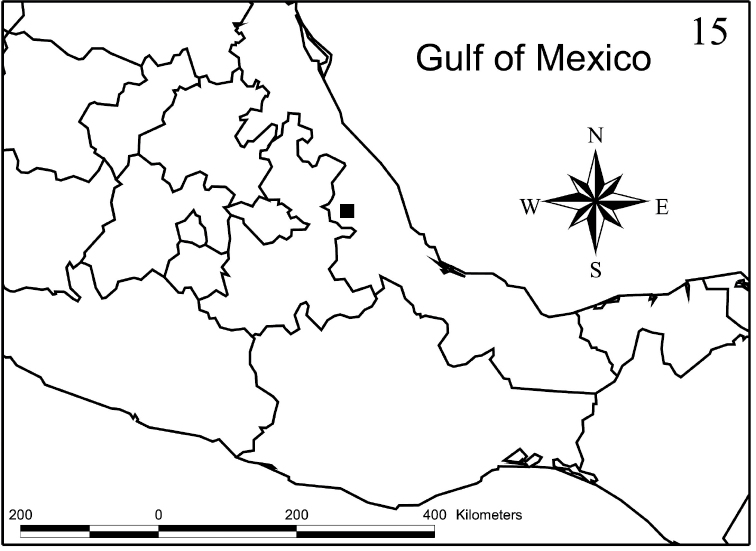
Collection site of *Placukorna ipsa* Santiago-Jiménez gen. n. and sp. n. (black square).

#### Distribution.

The single described species, *Placukorna ipsa*, is known only from the Cofre de Perote volcano, in the central region of Veracruz state, in Mexico. Apparently, the genus is distributed in montane areas.

#### Identification key to the genera of Placusini

**Table:** 

1	Head transverse; without neck (Fig. 4)	**2**
–	Head quadrate to slightly transverse or transversally sub-orbicular; with neck (fig. 9.155 in Navarrete-Heredia et al. 2002)	**3**
2	Male tergite VIII with a variable number of small to large spine–dents in the apex (figs 33–49 in Klimaszewski et al. 2001)	***Placusa* Erichson, 1837**
–	Male and female tergite VIII modified to form a curved horn (Figs 2 and 11)	***Placukorna* gen. n.**
3	Head transversally sub-orbicular; pronotum with distinct anterior angles, posterior angles indistinct, with a long transverse sulcus; abdomen strongly narrowed from base to apex (fig. 1 in Pace 2013)	***Speiraphallusa* Pace, 2013**
–	Head quadrate; pronotum with anterior angles broadly rounded, posterior angles distinct, without a long transverse sulcus; abdomen more or less parallel–sided to slightly widened (fig. 2 in Ashe and Kistner 1989; fig. 549 in Pace 2008)	***Euvira* Sharp, 1883 and *Kirtusa* Pace, 2008**

### Placukorna ipsa

Taxon classificationAnimaliaColeopteraStaphylinidae

Santiago-Jiménez sp. n.

http://zoobank.org/F82847FD-34C8-4AFA-B1C2-0B90A1930258

Figs 1 , 2 , 3–14

#### Type locality.

México: Veracruz, Perote, Ejido Agua de los Pescados, 3090 m a.s.l., 19°31'30"N, 97°07'00"W, mixed pine forest, Lindgren trap # 14, 08–15.V.2015, P. Domínguez, C. Ruíz and R. Santiago.

#### Type material.

Holotype, male, pinned. Original label: “México: Veracruz, Perote, Agua de los Pescados. B. Pino mixto, 19°31'30"N, 97°07'00"W, 3086 m, tr. Lindgren #14, 08.V–15.V.2015, P. Domínguez, C. Ruíz, R. Santiago”/ “MUZ-UV-COL-00003446”/ HOLOTYPE *Placukorna ipsa* Santiago-Jiménez, 2016” [red label].

#### Other material.

Paratypes, same data as holotype except for: tr. de cruz # 6 (1 specimen ♂); same data except for: tr. de cruz # 8 (1 specimen ♀); same data except for: tr. de cruz # 7, 20.III–27.III.2015 (1 specimen on slide ♂); same data except for: tr. Lindgren # 11, 27.III–03.IV.2015 (6 specimens: 2 ♀ on slide; 2 specimens ♂ and 2 specimens ♀); same data except for: tr. Lindgren # 13 (1 specimen ♂); same data except for: tr. Lindgren # 14 (2 specimens: 1 specimen ♀ and 1 specimen ♂); same data except for: tr. de cruz # 6 (2 specimens ♂); same data except for: tr. de cruz # 7 (2 specimens ♀); same data except for: tr. de cruz # 9 (1 specimen ♂); same data except for: tr. Lindgren # 14, 10.IV–17.IV.2015 (1 specimen ♂); same data except for: tr. de cruz # 8, 17.IV–24.IV.2015 (2 specimens: 1 specimen ♂ and 1 specimen ♀); same data except for: tr. Lindgren # 13 (1 specimen ♀); same data except for: tr. de cruz # 7, 24.IV–01.V.2015 (1 specimen ♂); same data except for: tr. Lindgren # 12 (1 specimen ♀); same data except for: tr. Lindgren # 14 (1 specimen ♂); same data except for: tr. de cruz # 9, 01.V–08.V.2015 (2 specimens ♀); same data except for: tr. Lindgren # 12, 15.V–22.V.2015 (1 specimen ♂). All specimens deposited in MUZ-UV under numbers MUZ-UV-COL-00003447 to 00003473 [yellow label].

#### Diagnosis.

Although for the moment it is the only one known species to the genus, it is distinguished by the following combination of characters: body length 2.5–3.0 mm; head and pronotum dark brown and abdomen black; apical half or apical third of sternites III-VI reddish brown; elytra dark brown to black; coxae dark brown to black; metatrochanter and metafemur dark brown or yellowish brown, remaining legs yellowish brown; antennomeres 1–11 dark brown; surface of tergites and sternites III–VII with reticulate microsculpture, less evident on tergites III–IV; tergites III–VII with basal impression, III–V almost straight, VI posteriorly slightly curved, VII posteriorly evidently curved; tergite VI with a protuberance on each side of midline; spermatheca with small and approximately spherical capsule, median portion of spermathecal stem (duct) narrowly U–shaped, and posterior portion also U–shaped, with accessory gland; median lobe with moderately large bulbus, tubus almost straight, internal sac of median lobe with evident spinules, apex blunt in lateral view, with short compressor plate (less than half of median lobe), basal ridge convex and pointed; flagellum short, without coils in bulbus; process of crista apicalis long, almost straight and parallel to median lobe, rounded at the apex.

#### Description.

Body length 2.5–3.0 mm. Head and pronotum dark brown and abdomen black; apical half or apical third of sternites III-VI reddish brown; elytra dark brown to black; coxae dark brown to black; metatrochanter and metafemur dark brown or yellowish brown, remaining legs yellowish brown. The apical edge of tergite III can be reddish.

*Head*. Dorsal surface without impression, protuberance or carina on disc (Fig. 4). Antennomeres 1–11 dark brown. Antennomeres 1–2 very long, 3 long, 4–10 transverse, and 11 long (Fig. 3).

*Mouthparts*. As described for the genus.

*Thorax*. As described for the genus.

*Abdomen*. As described for the genus. Other conspicuous characters are: surface of tergites and sternites III–VII with reticulate microsculpture, less evident on tergites III–IV; tergites III–VII with basal impression, III–V almost straight, VI posteriorly slightly curved, VII posteriorly evidently curved, and tergite VI with a protuberance on each side of midline.

*Secondary sexual structures*. There are differences between the sexes in the shape of sternite VIII and the number of macrosetae on it (Figs 8, 9). No other visible secondary sexual characters were found.

*Female*. Spermatheca with small and approximately spherical capsule, median portion of spermathecal stem (duct) narrowly U–shaped, and posterior portion also U–shaped, with accessory gland (Fig. 13).

*Aedeagus*. Median lobe with moderately large bulbus, tubus almost straight, internal sac of median lobe with evident spinules, apex blunt in lateral view, with short compressor plate (less than half of median lobe), basal ridge convex and pointed; flagellum short, without coils in bulbus (Fig. 12). Process of crista apicalis long, almost straight and parallel to median lobe, rounded at the apex (Fig. 12). Paramere with anterodorsal margin of paramerite with prominent sensory pores present beneath the velar sac (Fig. 14); hinge zone of paramerite evident, extended from dorsal surface to near articulation between condylite and paramerite; apical process of paramerite clearly articulated anterior to edge of velum; condylite with row of sensory pores; velum short (less than half length of paramere).

#### Remarks.

*Placukorna ipsa* is the only described species in the genus. The characters that could be useful at the species level are the shape of the aedeagus, spermatheca, and impressions and protuberances on the abdomen as described above.

Some characters that vary among the specimens collected are: protuberances on each side of midline of tergite VI are inconspicuous to prominent, one specimen had a raised midline from tergite III–VI, and the horn of tergite VIII is as long as tergite VII or tergites VI–VII together.

#### Etymology.

As the specimens were found associated with Scolytinae of the genera *Ips* DeGeer and *Pseudips* Cognato, the name makes reference to *Ips* from Greek “ἴψ” (meaning sort of worm), with Greek ending “a”.

#### Gender.

Neuter.

#### Habitat.

Specimens of *Placukorna* are possibly living in galleries of *Ips* and *Pseudips* associated with different *Pinus* species of the mixed pine forest (*Pinus pseudostrobus* predominating) where they were collected. Specimens were collected using traps baited with a mix of semiochemicals (ipsenol, ipsdienol and lanierone), in which more than 180 specimens of *Ips* (94) and *Pseudips* (91) were also collected. The semiochemicals are commonly used in those traps to capture bark beetles (Scolytine), therefore, an association of specimens of *Placukorna* with *Ips* and *Pseudips* is plausible.

#### Distribution.

*Placukorna ipsa* sp. n. is known from the type locality in the central region of Veracruz, Mexico. Twenty-eight specimens of *Placukorna ipsa* sp. n. were captured by handmade intercept traps for bark beetles primed with the semiochemicals mentioned above, in mixed pine forest. The locality Agua de los Pescados is 3090 m a.s.l. on the northeast face of the Cofre de Perote, Veracruz, Mexico (Fig. 15).

## Discussion

Tribe Placusini as proposed by Ashe (1991) has eight synapomorphies, of which two characters are unique to this tribe: mandible with dorsal molar area modified with transverse rows of large teeth, and a similar distribution of dorsal sensory pores on the mandible. Moreover, Placusini possess: a tarsal formula of 4–4–5; labrum rounded medially with a small a-sensillum; epipharynx with longitudinal medial field of small pores flanked on either side with row of large scales; with dorsal velvety patch modified to transverse rows of large teeth; without rows of denticles on molar region of ventral (condylar) side; labium with short, two-articled labial palpi; lateral pseudopores composed of two asetose and one setose pores, among other characteristics. Recently, a diagnosis of the genus *Placusa* was proposed by Klimaszewski et al. (2001), and they recognized 8 species of *Placusa* from Canada. Also, 8 species were mentioned from North America by Newton et al. (2000); whereas only two species have been described from Mexico, Pace (1990): *Placusa flohri* and *Placusa uhligi*, with a Neotropical distribution. *Placusa* can be distinguished from *Euvira* by the following characters: head transverse (in *Euvira* it is quadrate), without a distinct neck (distinct neck in *Euvira*), pronotum with distinct anterior angles (broadly rounded in *Euvira*), posterior angles indistinct (distinct in *Euvira*), base of pronotum evenly arcuate (strongly arcuate in *Euvira*); abdominal tergite VII not longer than VI (much longer in *Euvira*)(Ashe and Kistner 1989; Navarrete-Heredia et al., 2002). In contrast, *Kirtusa* is close to *Placusa* in the morphology of the mouthparts, but the body is convex and not flattened, the neck is very narrow, the temples are not sulcate, and the shape of the spermatheca is more similar to that of most of the species of the genus *Gyrophaena* than to that of the genus *Placusa* (Pace 2008). Pace (2008) however, said nothing about *Euvira*, and we found that some of the characters used to distinguish *Kirtusa* are shared with *Euvira* (e.g. distinct neck, spermatheca shape). *Speiraphallusa* can be distinguished from *Placusa* by its very convex body, the great length of the terminal article of the labial and maxillary palpi, the first meso- and metatarsomere very long and secondary sexual characters on the elytra of the male (Pace 2013).

The new genus proposed here matched most of the synapomorphies of Placusini proposed by Ashe (1991), although some characters are slightly different, such as: lateral or ε-sensilla present on the epipharynx, medial field of epipharynx not flanked by rows of large scales, and prementum with lateral field of pores with pseudopores extended from medial field. The new genus is very similar to *Placusa* and possesses several of the diagnostic characters proposed by Klimaszewski et al. (2001), except for the spine-like dents of tergite VIII. However, the new genus shows strong modifications on the male and female abdomen, mainly in tergite VIII. Male and female tergite VII is also modified to support the long horn of tergite VIII, such that tergite VII has lateral internal walls in the margin that apparently support tergite VIII. Also, tergite VII possesses a sclerotized internal plate in the posterior margin that is probably also supporting tergite VIII. This internal plate could be an intersegmental membrane between tergite VII and VIII that was sclerotized but more studies are necessary to understand this modification. All of these characteristics are useful for distinguishing the new genus from the other Placusini. That being said, we were not able to find any differences between the genera *Kirtusa* and *Euvira* when preparing the dichotomous key, so the key does not distinguish them. Pace (2008) discussed some of the differences between *Kirtusa* and *Placusa*, but said nothing about *Euvira*, even though *Euvira* is widely distributed in the Americas and *Kirtusa* is only known from Ecuador (Ashe and Kistner 1989; Pace 2008). We therefore think that *Kirtusa* is probably a junior synonym of *Euvira*; however, that determination is beyond the scope of this work.

## Supplementary Material

XML Treatment for Placukorna

XML Treatment for Placukorna ipsa
